# The status and future directions of comprehensive tobacco control policies for the 2020 Tokyo Olympic Games: A review

**DOI:** 10.18332/tid/105453

**Published:** 2019-04-01

**Authors:** Meng Li, Aoki Tada, Misaki Kiya, Reiko Okamoto

**Affiliations:** 1 Public Health Nursing Laboratory, Division of Health Sciences, School of Medicine, Osaka University, Suita, Japan

**Keywords:** tobacco control policies, Olympic Games, MPOWER

## Abstract

**INTRODUCTION:**

This review aims to clarify the status and future directions of comprehensive tobacco control policies for the 2020 Tokyo Olympic Games based on a comparison with seventeen countries that hosted the Olympic Games.

**METHODS:**

Seventeen countries that hosted the Olympic Games from 1988 to 2018 were identified by searching the website of IOC. A comparison of the contents was carried out by six themes (Monitor, Protect, Offer, Warn, Enforcement and Raise) in accordance with the MPOWER package for implementation of the FCTC. The comparison items and data were obtained from Global Health Observatory (GHO) data, Japanese government websites, and the literature. Based on the MPOWER scale, the 17 countries were ranked by total score, and a correlation between smoking prevalence and MPOWER total scores was established.

**RESULTS:**

The following three results were clarified: 1) Compared to the 17 countries that hosted the Olympic Games, smoking prevalence in Japan at 22.5% is at a moderate level and male smoking prevalence (33.7%) is still higher than other developed countries such as UK (24.7%) and Australia (16.5%); 2) Japanese tobacco control policies were far behind other countries that hosted the Olympic Games, especially in protecting people from tobacco smoke, warning about the dangers of tobacco, anti-tobacco mass media campaigns and enforcing bans on tobacco advertising, promotion, and sponsorship; and 3) The better practice in MPOWER was able to reduce total and male smoking prevalence in the study countries.

**CONCLUSIONS:**

Japanese tobacco control policies are far behind other countries that hosted the Olympic Games. In order to successfully hold the 2020 Tokyo Olympic Games, it will be necessary to strengthen tobacco control policies, based on the FCTC, in the future.

## INTRODUCTION

Tobacco use is a significant public health concern worldwide and a major cause of death and disability in both developed and developing countries^1^. Though tobacco use is steadily declining in developed countries, prevalence of smoking and cigarette consumption are increasing in many developing countries^2^. According to the 2017 World Health Organization (WHO) report, tobacco use is currently responsible for the death of about 7 million people worldwide each year^3^. It was also reported that tobacco use is also a major preventable factor for the development of non-communicable diseases (NCDs), including cancers, cardiovascular disease, diabetes, and chronic pulmonary obstructive disease (COPD)^4^. Therefore, it is necessary to promote and implement comprehensive tobacco control policies for preventing avoidable death and disability.

To reduce health problems due to smoking or passive smoking, WHO enacted the Framework Convention of Tobacco Control (FCTC) in the World Health Assembly, 21 May 2003, which entered into force on 27 February 2005. By December 2017, 181 countries worldwide had ratified the FCTC^4^. Furthermore, to facilitate country-level adoption and implementation of FCTC by the parties, WHO developed specific article implementation guidelines and created the MPOWER technical assistance package, which contains measures for monitoring FCTC progress to effectively manage and improve its implementation. The six components of MPOWER include: 1) Monitor tobacco use and prevention policies; 2) Protect people from tobacco smoke; 3) Offer help to quit tobacco use; 4) Warn about the dangers of tobacco; 5) Enforce bans on tobacco advertising, promotion and sponsorship; and 6) Raise taxes on tobacco^5^.

The Olympic Games are one of the world’s premier sporting events. In 1988, the Canadian National Olympic Committee (NOC) designated the Calgary Winter Games as the first ‘Smoke-Free’ Olympics, banning tobacco sponsorship and introducing non-smoking areas. After that, in July 2010, promotion of the Olympics as ‘tobacco-free’ was emphasized in an agreement between the WHO and the International Olympic Committee (IOC)^6^. The findings of a previous study^7^ suggest that for the Olympics to be completely tobacco-free, a comprehensive policy based on the FCTC should be adopted and enforced throughout the Olympic movement. Therefore, to successfully hold the 2020 Tokyo Olympic games, it is necessary to clarify Japanese status and future directions of comprehensive tobacco control policies based on a comparison with other countries that hosted the Olympic Games.

## METHODS

### Data sources

First, we identified 17 countries that hosted the Summer Olympic Games, Winter Olympic Games and Youth Olympic Games from 1988 to 2018 by searching the website of IOC (https://www.olympic.org/olympic-games). These 17 countries included South Korea, Brazil, Russia, England, Canada, China, Italy, Greece, Australia, Japan, United States, Norway, Spain, France, Argentina, Austria, and Singapore. Second, a comparison of the contents was carried out by six themes (Monitor, Protect, Offer, Warn, Enforcement and Raise) in accordance with the MPOWER package for implementation of the FCTC. The comparison items and data of each theme were obtained from Global Health Observatory (GHO) data. Third, the latest data on tobacco control polices in Japan, especially focusing on the 2020 Tokyo Olympics and Paralympic Games, were obtained from Japanese government websites such as Ministry of Health, Labor and Welfare, Ministry of Finance, Japan, Japan e-government and the literature.

### Analysis

First, the data of each theme from GHO were triangulated to categories by comparison items and review countries. Second, implementation status of each theme was identified by a score based on the MPOWER evaluation tool for FCTC implementation used by WHO, and 17 study countries were ranked by total score. Third, scatterplots were used to display the distribution of MPOWER total points and smoking prevalence, and Spearman’s correlation coefficients were used to test the correlation between smoking prevalence and MPOWER total points in the 17 countries. In addition, the correlation was considered to be significant if the association had a p<0.05. The SPSS Statistics 25.0 was used for the analysis.

## RESULTS

### Smoking prevalence

Of the 17 countries, smoking prevalence in Greece was the highest (43.7%), followed by the Russian Federation (40.9%) and France (32.9%), while smoking prevalence in Singapore, Australia, Canada, Brazil were less than 20 per cent (16.8%, 14.8%, 14.3%, 14.0%, respectively). Male smoking prevalence in Russia was higher than any other country (58.3%) followed by Greece (52.0%) and China (48.4%). Female smoking prevalence in Greece was higher than any other country (35.3%), followed by France (30.1%) and Austria (28.4%), while female smoking prevalence in China (1.9%) was lower than any other country in the study. Smoking prevalence in Japan was at a moderate (22.5%) (male 33.7%, female 11.7%) level of the 17 countries (Table 1).

**Table 1:** Smoking prevalence (%) among persons aged 15 years and older in seventeen countries that hosted the Olympic Games from 1988 to 2018

*Country*	*Total*	*Male*	*Female*
Greece	43.7	52.0	35.3
Russian Federation (Russia)	40.9	58.3	23.4
France	32.9	35.6	30.1
Austria	29.7	30.9	28.4
Spain	29.4	31.4	27.4
China	25.2	48.4	1.9
Italy	23.8	27.8	19.8
Republic of Korea (Korea)	23.6	40.9	6.2
Japan	22.5	33.7	11.2
United Kingdom of Great Britain and Northern Ireland (UK)	22.4	24.7	20.0
Argentina	22.0	27.7	16.2
United States of America (USA)	21.9	24.6	19.1
Norway	20.2	20.7	19.6
Singapore	16.8	28.3	5.2
Australia	14.8	16.5	13.0
Canada	14.3	16.6	12.0
Brazil	14.0	17.9	10.1

Last updated: 2018-03-23

### Summary of MPOWER measures

Except for China and Brazil assessed at 3 points, the other 15 countries were assessed at 4 points in monitoring tobacco use and prevention policies. Eight countries (UK, Australia, Argentina, Brazil, Canada, Russia, Spain, Greece) were assessed at 5 points, the highest level, while France and Italy were assessed at 1 point, in protecting people from tobacco smoke. Eight countries (UK, Australia, Argentina, Brazil, Singapore, Canada, USA, Korea) were assessed at 5 points, the highest level, while 9 countries were assessed at 4 points, in offering help to quit tobacco use. Five countries (Australia, Argentina, Brazil, Singapore, Canada) were assessed at 5 points in warning about the dangers of tobacco. Eight countries (UK, Australia, Singapore, Norway, Russia, USA, Korea, China) were assessed at 5 points, while Greece was assessed at 1 point in anti-tobacco mass media campaigns. Brazil, Russia and Spain were assessed at 5 points, while Korea and USA were assessed at 2 points in enforcing bans on tobacco advertising, promotion and sponsorship. Five countries (UK, Spain, France, Greece, Italy) were assessed at 5 points, the highest level, in raising taxes on tobacco. The detailed items and data of each MPOWER theme are shown in Table 1.

Finally, the 17 countries were ranked by MPOWER total scores (Table 2). Only 5 countries (UK, Australia, Argentina, Brazil, Singapore) were assessed to have 30 points or more, while the total score for Japan was 21, the lowest, especially in protecting people from tobacco smoke (2 points), warning about the dangers of tobacco (3 points), anti-tobacco mass media campaigns (2 points) and enforcing bans on tobacco advertising, promotion, and sponsorship (2 points).

**Table 2:** Seventeen countries that hosted the Olympic Games, from 1988 to 2018, ranked by MPOWER evaluation tool

	*M*	*P*	*O*	*W*		*E*	*R*	*TOTAL*
	*Monitoring tobacco use and prevention policies*	*Protecting people from tobacco smoke*	*Offering help to quit tobacco use*	*Warning about the dangers of tobacco*	*Anti-tobacco mass media campaigns*	*Enforcing bans on tobacco advertising, promotion and sponsorship*	*Raising taxes on tobacco*	
UK	4	5	5	4	5	4	5	32
Australia	4	5	5	5	5	4	4	32
Argentina	4	5	5	5	4	4	4	31
Brazil	3	5	5	5	4	5	4	31
Singapore	4	3	5	5	5	4	4	30
Canada	4	5	5	5	2	4	4	29
Norway	4	4	4	4	5	4	4	29
Russia	4	5	4	3	5	5	3	29
Spain	4	5	4	4	2	5	5	29
France	4	1	4	4	4	4	5	26
Greece	4	5	4	3	1	4	5	26
USA	4	2	5	4	5	2	3	25
Italy	4	1	4	3	4	4	5	25
Korea	4	2	5	3	5	2	4	25
China	3	2	4	3	5	4	3	24
Austria	4	2	4	3	2	4	4	23
Japan	4	2	4	3	2	2	4	21

Last updated: 2015-08-31

### The correlation between smoking prevalence and MPOWER total scores

**Figure 1:**
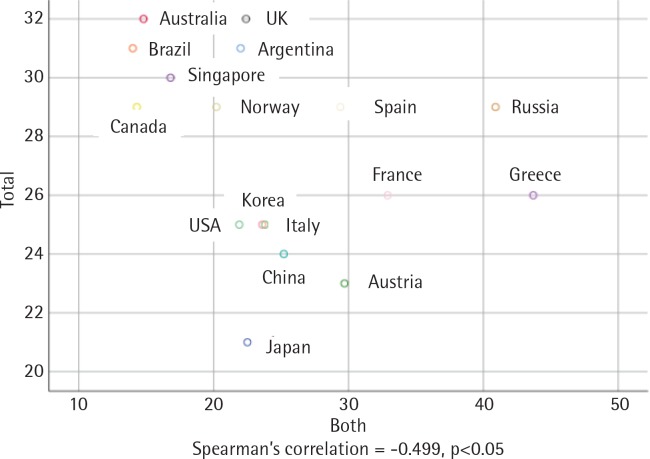
Scatterplot of smoking prevalence and MPOWER total points

**Figure 2:**
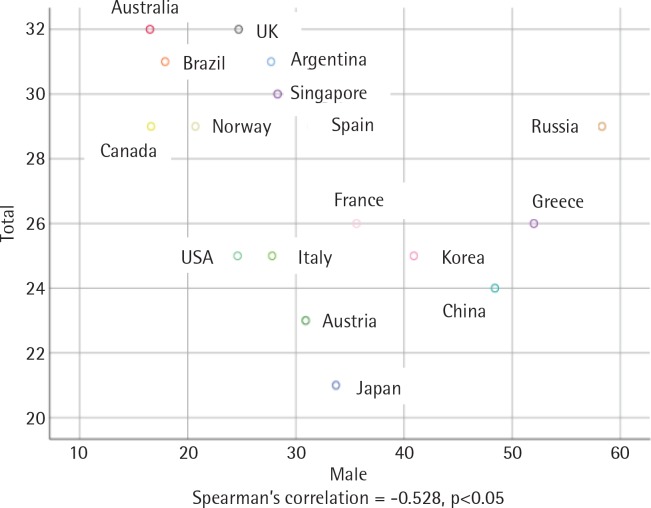
Scatterplot of male’s smoking prevalence and MPOWER total points

**Figure 3:**
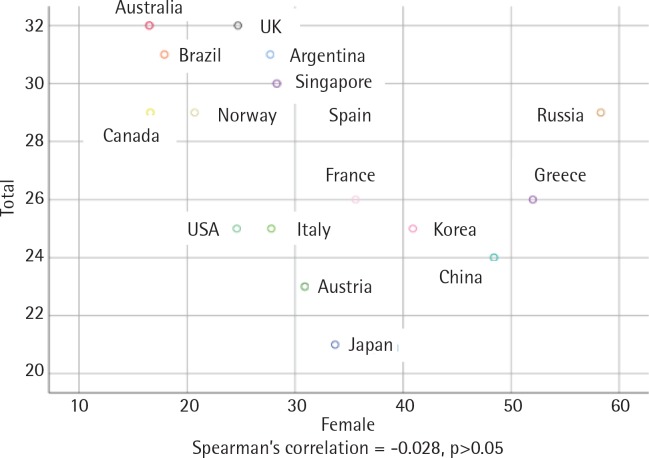
Scatterplot of female’s smoking prevalence and MPOWER total points

There was an inverse and statistically significant correlation between smoking prevalence and MPOWER total scores (r = -0.499, p<0.05) (Figure 1). The correlation between male smoking prevalence and MPOWER total scores was positive and statistically significant (r = -0.528, p<0.05) (Figure 2), while the correlation between female smoking prevalence and MPOWER total scores was not statistically significant (r = -0.028, p>0.05) (Figure 3). Overall, the higher MPOWER total scores are, the lower both smoking prevalence and male smoking prevalence are.

## DISCUSSION

From the results, it is obvious that Japanese tobacco control policies are far behind other countries that hosted the Olympic Games, especially in protecting people from tobacco smoke, warning about the dangers of tobacco, anti-tobacco mass media campaigns and enforcing bans on tobacco advertising, promotion, and sponsorship.

### Monitoring smoking prevalence

The current adult smoking prevalence in Japan is 22.5% (GHO data), a moderate level amongst the 17 countries. Male smoking prevalence (33.7%) in Japan is still higher than other developed countries such as UK (24.7%) and Australia (16.5%), although it is reported that smoking prevalence of adult men has continued to decline in the past few decades in Japan8. Female smoking prevalence (11.2%) in Japan is remarkably higher than the other two East Asia countries, China 1.2% and Korea 6.2%. Concerning the goals of tobacco control in the future, Health Japan 21 (2nd) adopted by Ministry of Health, Labor and Welfare in 2013, set four goals for 2022: 1) the decrease of adult smoking rate (19.5% → 12%); 2) the decrease of minors’ smoking (male: 8.6% → 0%, female: 3.8% → 0%); 3) the decrease of smoking during pregnancy (5.0% → 0%); and 4) the decrease of passive smoking (home, workplace, restaurant, government agency, medical institution)^9^. In order to achieve these goals, it is necessary to implement comprehensive tobacco control measures.

### Protecting people from tobacco smoke

In order to protect people from tobacco smoke, Article 8.2 of FCTC^10^ states that ‘Each party shall adopt and implement in areas of existing national jurisdiction as determined by national law and actively promote at other jurisdictional levels the adoption and implementation of effective legislative, executive, administrative and/or other measures, providing for protection from exposure to tobacco smoke in indoor workplaces, public transport, indoor public places and, as appropriate, other public places’. Concerning the implementation of smoke free policies, Ireland, in 2004, was first to enact comprehensive smoke free laws in the world, not only in the general workplace but also in all indoor facilities including restaurants and bars, before FCTC came into force in 2005. Furthermore, similar laws were enacted in many countries and regions including New Zealand (2004), Uruguay (2006), UK (2007), Hong Kong, Turkey (2009) and more than half of the states in USA11. By 2016, 55 countries had enacted similar comprehensive smoke free laws, even in some developing countries^12^.

In Japan, Article 25 of ‘Health Promotion Law’^13^ enforced in 2003 states that in order to prevent passive smoking, national and local public organizations have an obligation to comprehensively and effectively promote the measures of spreading knowledge on passive smoking, raising awareness on prevention of passive smoking, improving the environment and promoting other measures in prevention of passive smoking. In addition, ‘Industrial Safety and Health Law’^14^ enforced in 2015 states that managers of facilities have an obligation to promote smoke free indoor places and separate smoking areas. Moreover, ‘Health Promotion Law’ and ‘Industrial Safety and Health Law’ have not set penalties for the violators. At the prefectural level, two ordinances preventing passive smoking in public places, with penalties, were enforced in Kanagawa Prefecture^15^ and Hyogo Prefecture^16^, but two ordinances didn’t mandate a complete smoke-free environment in public places, and did not cover all indoor places.

Recently, with the 2020 Tokyo Olympic Games approaching, to strengthen measures to prevent passive smoking in a wide range of public places, a ‘Passive smoking prevention measures reinforcement study team’, chaired by the Deputy Secretary of the Cabinet Secretariat, was established in 2016, and the Japanese government began to discuss strengthening smoke free policies^12^. Furthermore, the Tokyo Metropolitan government passed the ‘Passive Smoking Prevention Ordinance’ on 27 June 2018, and the ordinance will be fully enforced in 1 April 2020^17^. Like the ordinances of Kanagawa and Hyogo, the Tokyo ordinance was also implemented with penalties, however, Tokyo ordinance also didn’t mandate a complete smoke-free environment in public places, and did not cover all indoor places. Overall, it is expected that a national tobacco free law will be established that can cover the whole country in all indoor facilities with penalties, instead of self-regulation by facility managers, in the future.

### Offering help to quit tobacco use

In order to offer help to quit tobacco use, Article 14.1 of FCTC^10^ states that ‘each party shall develop and disseminate appropriate, comprehensive and integrated guidelines based on scientific evidence and best practices, taking into account national circumstances and priorities, and shall take effective measures to promote smoking cessation and adequate treatment for tobacco dependence’. In Japan, ‘Basic Plan to Promote Cancer Control Programs’18 adopted in June 2012 by the Cabinet Office, states that all smokers who want to quit smoking will be given nicotine dependence treatment to help them quit smoking. Concerning the implementation of smoking cessation support, the Japanese smoking cessation therapy (SCT) covered by the medical insurance system in Japan started in April 2006, and nicotine patch and varenicline were introduced as smoking cessation medication in April 2006 and May 2008, respectively. After then, nicotine substitute patch and gum were switched to over-the-counter (OTC), so it became possible to purchase them in a general store without a prescription. The first edition of smoking cessation guidelines was published in 2006, and the sixth edition was revised in 2014 based on the latest data on the effectiveness of smoking cessation supplements^19^. In addition, the content of smoking cessation guidance in ‘Standard Health Examination/ Health Guidance Program [Revised Edition]’ was strengthened, and to be easily used by community health workers and workplace hygiene managers, basic knowledge on tobacco and smoking cessation methods for health education were added into the new ‘Smoking Cessation Support Manual’ in 2013^11^.

In all, the Japanese smoking cessation therapy system has made great progress in the last decade, but a toll-free quitline for smoking cessation has not been established. Therefore, it is necessary to set up a toll-free quitline with free access in the future.

### Warning about the dangers of tobacco

In order to warn about the dangers of tobacco, Article 11.1(b) of FCTC^10^ states that warnings/messages required the use of pictures or photos on cigarette packages and the size of warnings should be 50% or more of the principal display areas but shall be no less than 30% of the principal display areas. Concerning the implementation of health warning pictures or photos, Canada was the first country to use health warning pictures or photos in 2000, after then, Australia, Europe, Africa, Thailand, Malaysia, Philippines and other South-East Asian countries also gradually implemented this measure. In 2010, there were 34 countries that used health warning pictures or photos, which increased to 63 countries in 2014^25^. In Japan, the warning message ‘Be careful of smoking too much for your health’ was for the first time displayed on the side of cigarettes in 1972, and the warning message ‘since smoking might damage your health, be careful of smoking too much’ has been displayed since 1990. However, the two warning messages just presented the view that if one does not smoke too much then smoking will not damage one’s health. Currently, it is obligatory to display two types of warning texts^25^ on two sides of the tobacco packaging according to Article 36 of Tobacco Business Law^23^. However, the display areas of warning messages are only 30% of the front and 30% of the back, and so only the minimum requirement of FCTC is satisfied.

In addition to health warning pictures or photos, Article 11.1(a) of FCTC^10^ states that tobacco product packaging and labeling must not promote a tobacco product by any means that are false, misleading, deceptive or likely to create an erroneous impression about its characteristics, health effects, hazards or emissions, including any term, descriptor, trademark, figurative or any other sign that directly or indirectly creates the false impression that a particular tobacco product is less harmful than other tobacco products. These include terms such as ‘low tar’, ‘light’, ‘ultra-light’, or ‘mild’. In addition to the above terms, in some South-East Asian countries such as Malaysia and Thailand, ‘cool’, ‘extra’, ‘special’, ‘smooth’, ‘premium’, ‘natural’ were also cited as prohibited terms^25^. The European Union Member States are obliged not to use these terms in accordance with domestic rules and law, and legislation to prohibit these terms was also planned based on EU laws. In Japan, according to Article 36(2) of the Tobacco Business Law^23^, if the meaning of terms such as ‘mild’ and ‘light’ do not refer to degree of tobacco taste, and are mildly influential on health, then these terms can be continued to be used.

In addition, guidelines for the implementation of Article 1126 state that parties should prohibit the display of figures for emission yields (such as tar, nicotine, and carbon monoxide) on packaging and labeling, when used as part of a brand name or trademark. However, in Japan, as part of the means to provide consumers objective information related to smoking and health, the amount of nicotine and tar should be displayed numerically on cigarette packaging according to the Tobacco Business Law^23^. Furthermore, guidelines for the implementation of Article 1126 also state that parties should consider adopting measures to restrict or prohibit the use of logos, colors, brand images or promotional information on packaging other than brand names and product names, displayed in a standard colour and font style. However, in Japan, standard colour and font style on packaging (plain package) were not implemented.

In the future, it will be expected that promoting the use of health warning pictures or photos, prohibiting the display of some misleading terms and figures for emission yields and standard colour and font style on packaging will be regulated by law.

### Enforcing bans on tobacco advertising, promotion and sponsorship

In order to enforce bans on tobacco advertising, promotion and sponsorship, Article 13.2 of FCTC^10^ states that ‘each party shall, in accordance with its constitution or constitutional principles, undertake a comprehensive ban of all tobacco advertising, promotion and sponsorship’. In Japan, Tobacco Business Law^23^ enacted by the Ministry of Finance on 10 August 1984 states that tobacco advertisement should not be excessive. ‘Guidelines for advertisement on manufactured tobacco’^24^ enacted by the Ministry of Finance in 1989 and revised in March 2004 just states that whether individuals choose to smoke is a personal judgment based on the consideration of individual responsibility. In addition, ‘self-regulation criteria on tobacco advertising, promotion activities and packaging of manufactured cigarettes’^27^ was enacted by the Tobacco Institute of Japan in 1989 and revised in 2007. Tomofumi^28^ summarized characteristics of these self-regulation criteria including: 1) the self-regulation criteria were not applied to corporate advertisements, improving smoking manners advertising and prevention of minors smoking advertising; 2) the scope of tobacco advertising and sponsorship regulations for minors were not thorough; and 3) Corporate Social Responsibility (CSR) activities were not mentioned.

Overall, tobacco advertising, promotion and sponsorship in Japan were not prohibited by law, and only depended on the enterprise’s self-regulation but the self-regulation was not thorough. Therefore, It is expected that tobacco advertising, promotion and sponsorship will be regulated by an explicit law in the future.

### Raising taxes on tobacco

In order to raise taxes on tobacco, Article 6.2 of FCTC^10^ states that the parties recognize that price and tax measures are an effective and important means of reducing tobacco consumption by various segments of the population, in particular young persons. In Japan, since a special tobacco tax was established in 1998, tobacco tax was increased by 8% in 2003 and by 11% in 2006, respectively, resulting in a price increase of 1 Yen per cigarette. In addition, tobacco tax increased by 37% in 2010, resulting in a price increase of 3.5 Yen per cigarette, considered as the biggest tax/price rise in the past^29^. Although the tobacco price has substantially increased in the last decade, the total tax in Japan (average tobacco tax, 62.9%; most sold brands of cigarettes, 64.4%) is still very low, and the tobacco price is less than half of that in other developed countries such as the UK and Australia. Therefore, it is expected that there will be further increases in tobacco tax in the future.

## CONCLUSIONS

Comprehensive tobacco control polices have been implemented in many countries with the entry into force of the FCTC since 27 February 2005. However, tobacco control policies in Japan are far behind other countries that hosted Olympic Games, especially in protecting people from tobacco smoke, warning about the dangers of tobacco, anti-tobacco mass media campaigns, and enforcing bans on tobacco advertising, promotion and sponsorship. In the future, it is expected that a national tobacco-free law will be implemented to cover the whole country in all indoor facilities with penalties instead of self-regulation by facility managers, to set up a toll-free quitline with free access, to use standard colour and font style on packaging, to regulate tobacco advertising, promotion and sponsorship, and to further increase tobacco tax. We have clarified and also stress that since the better practice of MPOWER was able to reduce total and male smoking prevalence in the 17 countries that hosted Olympic Games from 1988 to 2018, it will be necessary to further strengthen tobacco control policies in Japan based on the FCTC in order to successfully hold the 2020 Tokyo Olympic Games.
