# Supplementary material for: The status and future directions of comprehensive tobacco control policies for the 2020 Tokyo Olympic Games: A review
Source: Tob Induc Dis. 2019 Apr 1;17:24. doi: 10.18332/tid/105453 (PMC6751969; doi:10.18332/tid/105453)
Supplement: Supplementary file 1 [file TID-17-24-s1.pdf]

Table 3 Protecting people from tobacco smoke, tobacco advertising, promotion and sponsorship in 17 hosting Olympic games countries

	Argentina	Brazil	Canada	USA	Austria	France	Greece	Italy	Norway	Spain	UK	Australia	China	Japan	Korea	Singapore	Russia
**Protecting people from tobacco smoke (Not Applicable = NA)**																	
Number of places smoke-free	8	8	8	1	1	3	8	0	7	8	8	8	1	0	2	5	8
Fines for violations	Yes	Yes	Yes	No	Yes	Yes	Yes	Yes	Yes	Yes	Yes	No	No	No	Yes	Yes	Yes
Fines on the establishment	Yes	Yes	Yes	NA	Yes	Yes	Yes	Yes	Yes	Yes	Yes	NA	NA	NA	Yes	Yes	Yes
Fines on the patron	No	No	Yes	NA	Yes	Yes	Yes	Yes	Yes	Yes	Yes	NA	NA	NA	Yes	Yes	Yes
Dedicated funds for enforcement	Yes	No	Yes	No	No	No	No	No	No	No	No	No	No	No	Yes	No	No
Citizen complaints and investigations	Yes	Yes	Yes	No	Yes	No	Yes	Yes	No	Yes	Yes	No	No	No	No	Yes	No
Subnational smoking bans - authority exists	Yes	Yes	Yes	Yes	Yes	No	No	No	No	Yes	Yes	Yes	Yes	Yes	No	NA	No
Comprehensive bans in place subnationally	No	No	Yes	Yes	No	NA	NA	NA	NA	No	Yes	Yes	Yes	No	NA	NA	NA
																	Last updated: 2015-09-02
**Tobacco advertising (Banned = Yes, Allowed = No)**																	
National TV and radio	Yes	Yes	Yes	Yes	Yes	Yes	Yes	Yes	Yes	Yes	Yes	Yes	Yes	No	Yes	Yes	Yes
International TV and radio (broadcast from abroad, including satellite)	Yes	Yes	Yes	Yes	Yes	Yes	Yes	Yes	Yes	Yes	Yes	Yes	Yes	No	Yes	Yes	Yes
Local magazines and newspapers	Yes	Yes	No	No	Yes	No	Yes	Yes	No	Yes	Yes	Yes	Yes	No	Yes	Yes	Yes
International magazines and newspapers	Yes	Yes	Yes	No	Yes	Yes	Yes	Yes	Yes	Yes	Yes	Yes	Yes	No	No	Yes	Yes
Billboards and outdoor advertising	Yes	Yes	No	No	Yes	No	No	No	No	No	No	No	Yes	No	No	Yes	Yes
Point of sale	Yes	Yes	Yes	No	Yes	Yes	Yes	Yes	Yes	Yes	Yes	Yes	No	No	Yes	Yes	Yes
Internet	No	Yes	No	No	No	No	No	Yes	Yes	Yes	No	No	No	No	No	Yes	Yes
**Promotion and sponsorship (Banned = Yes, Allowed = No)**																	
Free distribution of tobacco products in the mail or through other means	Yes	Yes	Yes	No	No	Yes	Yes	No	Yes	Yes	Yes	No	No	No	No	Yes	Yes
Promotional discounts	Yes	Yes	Yes	No	Yes	Yes	No	No	Yes	Yes	Yes	No	No	No	No	Yes	Yes
Non-tobacco goods and services identified with tobacco brand names	Yes	Yes	No	No	No	Yes	No	No	No	Yes	Yes	Yes	No	No	No	No	Yes
Brand name of non-tobacco products used for tobacco product	Yes	Yes	No	No	No	No	No	No	Yes	Yes	Yes	No	No	No	No	No	Yes
appearance of tobacco brands in TV and/or films (product placement)	Yes	Yes	Yes	No	No	Yes	Yes	No	Yes	Yes	Yes	No	No	No	No	Yes	Yes
																	Last updated: 2015-08-19

Table 4 Offer help to quit tobacco use in 17 hosting Olympic games countries

	Argentina	Brazil	Canada	USA	Austria	France	Greece	Italy	Norway	Russia	Spain	UK	Australia	China	Japan	Korea	Singapore
**Treatment for tobacco dependence (Fully = F, Partially= P, Yes, in some = Yes, is, Yes, in most= Yes, im, Not Applicable = NA)**																	
Access to a toll-free quit line	Yes	Yes	Yes	Yes	Yes	No	No	Yes	Yes	Yes	No	Yes	Yes	No	No	Yes	Yes
Treatment for tobacco dependence available in hospitals	Yes, im	Yes, is	Yes, im	Yes, is	Yes, is	Yes, im	Yes, is	Yes, is	Yes, is	Yes, is	Yes, is	Yes, im	Yes, im	Yes, is	Yes, is	Yes, is	Yes, im
Treatment for tobacco dependence in hospitals cost covered	F	F	P	P	P	P	P	P	F	No	F	F	P	P	P	P	P
Treatment for tobacco dependence available in offices of health professionals	Yes, is	Yes, im	Yes, im	Yes, is	Yes, is	Yes, is	Yes, is	Yes, is	Yes, is	Yes, is	Yes, is	Yes, im	Yes, im	Yes, is	No	NA	Yes, is
Treatment for tobacco dependence in offices of health professionals cost covered	P	No	P	P	P	P	P	No	No	F	F	F	P	P	NA	NA	P
Treatment for tobacco dependence available in the community	Yes, is	Yes, is	Yes, is	Yes, is	Yes, is	NA	No	Yes, is	Yes, is	No	Yes, is	Yes, im	Yes, is	Yes, is	Yes, is	NA	Yes, is
Treatment for tobacco dependence in the community cost covered	P	No	No	P	NA	NA	NA	No	No	NA	P	F	NA	No	P	NA	P
Treatment for tobacco dependence available in other settings	No	Yes, is	Yes, is	No	Yes, is	Yes, is	Yes, is	Yes, is	Yes, is	Yes, is	Yes, is	Yes, is	Yes, is	No	No	NA	Yes, is
Treatment for tobacco dependence in other settings cost covered	NA	No	P	NA	P	P	P	P	P	F	No	F	P	NA	NA	NA	P
Treatment for tobacco dependence available in health clinics or other primary care facilities	Yes, im	Yes, is	Yes, im	Yes, is	Yes, is	Yes, is	Yes, is	Yes, is	Yes, is	Yes, is	Yes, is	Yes, im	Yes, im	Yes, is	Yes, is	Yes, is	Yes, im
Treatment for tobacco dependence in health clinics or other primary care facilities cost covered	F	F	P	P	P	P	P	P	P	F	F	F	P	P	P	P	P

Last updated: 2015-09-02

	Argentina	Brazil	Canada	USA	Austria	France	Greece	Italy	Norway	Russia	Spain	UK	Australia	China	Japan	Korea	Singapore
Medication **(Fully = F, Partially= P, In a pharmacy without a prescription = P wo p, In a general store without a prescription = Gs wo p, In a pharmacy with a prescription = P w p)**																	
Nicotine replacement therapy - legally sold	Yes	Yes	Yes	Yes	Yes	Yes	Yes	Yes	Yes	Yes	Yes	Yes	Yes	Yes	Yes	Yes	Yes
Nicotine replacement therapy - place available	P wo p	P wo p	P wo p	Gs wo p	P wo p	P wo p	P wo p	P wo p	P wo p	P wo p	P wo p	Gs wo p	Gs wo p	P wo p	P wo p	P wo p	P wo p
Nicotine replacement therapy - cost covered	P	F	P	P	No	P	No	No	No	No	No	F	P	No	P	P	P
Nicotine replacement therapy on the Essential Medicines List	No	Yes	No	NA	No	Yes	No	No	No	Yes	Yes	NA	Yes	No	Yes	No	No
Bupropion - legally sold	Yes	Yes	Yes	Yes	Yes	Yes	Yes	Yes	Yes	No	Yes	Yes	Yes	Yes	No	Yes	Yes
Bupropion - place available	P w p	P w p	P w p	P w p	P w p	P w p	P w p	P w p	P w p	NA	P w p	P w p	P w p	P w p	NA	P w p	P w p
Bupropion - cost covered	P	F	P	P	No	No	No	No	No	NA	No	F	P	No	NA	P	No
Varenicline - legally sold	Yes	Yes	Yes	Yes	Yes	Yes	Yes	Yes	Yes	Yes	Yes	Yes	Yes	Yes	Yes	Yes	Yes
Varenicline - place available	P w p	P w p	P w p	P w p	P w p	P w p	P w p	P w p	P w p	P w p	P w p	P w p	P w p	P w p	P w p	P w p	P w p
Varenicline - cost covered	P	No	P	P	No	No	No	No	No	No	No	F	P	No	P	P	No

Last updated: 2015-09-11

Table 5　Warn about the dangers of tobacco in 17 hosting Olympic games countries

	Argentina	Brazil	Canada	USA	Austria	France	Greece	Italy	Norway	Russia	Spain	UK	Australia	China	Japan	Korea	Singapore
**Health warning labels on cigarette packages**																	
Law mandates that health warnings appear on tobacco packages	Yes	Yes	Yes	Yes	Yes	Yes	Yes	Yes	Yes	Yes	Yes	Yes	Yes	Yes	Yes	Yes	Yes
Percentage of principal display area mandated to be covered by health warnings - front and back of cigarette packaging	50	65	75	50	35	35	35	35	35	40	35	35	82.5	30	30	30	50
Percentage of principal display area mandated to be covered by health warnings - front of cigarette packaging	50	30	75	50	30	30	30	30	30	30	30	30	75	30	30	30	50
Percentage of principal display area mandated to be covered by health warnings - back of cigarette packaging	50	100	75	50	40	40	40	40	40	50	40	40	90	30	30	30	50
Does the law mandate specific health warnings on cigarette packaging?	Yes	Yes	Yes	Yes	Yes	Yes	Yes	Yes	Yes	Yes	Yes	Yes	Yes	Yes	Yes	Yes	Yes
How many health warnings are approved by the law for cigarette packaging?	10	9	16	9	16	16	16	16	16	13	16	16	14	3	8	1	6
Do health warnings appear on each cigarette package and any outside packaging and labeling used in the retail sale?	Yes	Yes	Yes	Yes	Yes	Yes	Yes	Yes	Yes	No	Yes	Yes	Yes	Yes	Yes	No	Yes
Do health warnings on cigarette packaging describe the harmful effects of tobacco use on health?	Yes	Yes	Yes	Yes	Yes	Yes	Yes	Yes	Yes	Yes	Yes	Yes	Yes	Yes	Yes	Yes	Yes
Does the law mandate font style, font size and colour of health warnings on cigarette packaging?	Yes	Yes	Yes	Yes	Yes	Yes	Yes	Yes	Yes	Yes	Yes	Yes	Yes	Yes	No	Yes	Yes
Are the health warnings on cigarette packaging rotating?	Yes	Yes	Yes	Yes	Yes	Yes	Yes	Yes	Yes	Yes	Yes	Yes	Yes	Yes	Yes	Yes	Yes
Are the health warnings on cigarette packaging written in the principal language(s) of the country?	Yes	Yes	Yes	Yes	Yes	Yes	Yes	Yes	Yes	Yes	Yes	Yes	Yes	Yes	Yes	Yes	Yes
Do the health warnings on cigarette packaging include a photograph or graphic?	Yes	Yes	Yes	No	No	Yes	No	No	Yes	Yes	Yes	Yes	Yes	No	No	No	Yes

Last updated: 2015-08-14

	Argentina	Brazil	Canada	USA	Austria	France	Greece	Italy	Norway	Russia	Spain	UK	Australia	China	Japan	Korea	Singapore
**Anti-tobacco mass media campaigns (Not Applicable = NA)**																	
At least one national mass media campaign ran during the survey period	Yes	Yes	NA	Yes	NA	Yes	NA	Yes	Yes	Yes	NA	Yes	Yes	Yes	NA	Yes	Yes
Campaign was part of a comprehensive tobacco control programme	Yes	Yes	NA	Yes	NA	Yes	NA	Yes	Yes	Yes	NA	Yes	Yes	Yes	NA	Yes	Yes
Campaign was pre-tested	No	No	NA	Yes	NA	No	NA	No	Yes	Yes	NA	Yes	Yes	Yes	NA	No	Yes
Formative research was conducted	Yes	Yes	NA	Yes	NA	Yes	NA	Yes	Yes	Yes	NA	Yes	Yes	No	NA	Yes	Yes
Campaign aired on television and/or radio	Yes	No	NA	Yes	NA	Yes	NA	Yes	Yes	Yes	NA	Yes	Yes	Yes	NA	Yes	Yes
Campaign utilized media planning	Yes	Yes	NA	Yes	NA	Yes	NA	Yes	Yes	Yes	NA	Yes	Yes	Yes	NA	Yes	Yes
Earned media/public relations were used to promote the campaign	No	Yes	NA	Yes	NA	Yes	NA	No	Yes	Yes	NA	Yes	Yes	Yes	NA	Yes	Yes
Process evaluation was employed to assess implementation	Yes	Yes	NA	Yes	NA	Yes	NA	No	Yes	Yes	NA	Yes	Yes	Yes	NA	Yes	Yes

Last updated: 2015-08-19

Table 6 Raise taxes on tobacco in 17 hosting Olympic games countries

	Most sold brand of cigarettes - price in international dollars ($)	Average - cigarette price in international dollars ($)	Most sold brand of cigarettes - taxes as a % of price - total tax	Average - taxes as a % of cigarette price - total tax	Cigarettes are less affordable in 2014 compared to 2008
Greece	5.95	5.75	80.0	81.6	Yes
UK	11.01	11.2	82.2	81.3	Yes
France	7.76	7.61	80.3	80.6	Yes
Spain	6.82	6.39	78.1	78.8	Yes
Italy	5.82	5.43	75.7	76.1	Yes
Austria	5.63	5.34	74.0	74.9	Yes
Singapore	12.31	11.04	66.2	73.8	No
Argentina	3.42	3.47	69.8	69.7	Yes
Norway	8.99	9.31	68.8	67.2	Yes
Canada	7.5	6.88	69.8	64.5	Yes
Brazil	2.75	2.85	64.9	64.3	Yes
Japan	4.22	4.34	64.4	62.9	Yes
Korea	3.14	3.24	62.0	60.3	No
Australia	10.99	10.64	56.8	58.4	Yes
Russia	2.42	2.18	47.6	50.5	Yes
China	2.33	2.13	44.4	47.8	Yes
USA	6.23	6.07	42.5	42.9	Yes

Last updated: 2015-09-04

© 2019 Li M.
